# A. V. M. A.

**Published:** 1901-08

**Authors:** 


### A. V. M. A.

The following special rates have been secured at the New Hotel Rudolf: For headquarters and all members, $2.50 per day and upward for one person; $5 per day and upward for two persons in one room. These prices include the use of hot and cold sea-bath, which are located on each floor. Room with private bath attached, one person, $5 per day; room with private bath attached, two persons, $8 per day.

*Special Notice.* Those desiring to secure accommodations in advance at the Rudolf will please communicate direct to the hotel, giving the number of rooms required, number of persons to occupy the same, and if with private bath attached, and mentioning that they are delegates or members of the Society.

